# Influence of Blood Rheology and Turbulence Models in the Numerical Simulation of Aneurysms

**DOI:** 10.3390/bioengineering10101170

**Published:** 2023-10-08

**Authors:** Alberto Brambila-Solórzano, Federico Méndez-Lavielle, Jorge Luis Naude, Gregorio Josué Martínez-Sánchez, Azael García-Rebolledo, Benjamín Hernández, Carlos Escobar-del Pozo

**Affiliations:** 1 Thermofluids Department, Faculty of Engineering, UNAM, Coyoacan, Mexico City C.P. 04510, Mexicofmendez@unam.mx (F.M.-L.); jorge_naude@hotmail.com (J.L.N.); gregorio_martinez@comunidad.unam.mx (G.J.M.-S.); 2 Faculty of Mechanical and Electrical Engineering, Carretera Km 9 Colima-Coquimatlan, Colima C.P. 28400, Mexico; 3 Oak Ridge Leadership Computing Facility, Oak Ridge National Laboratory, Oak Ridge, TN 37830, USA; benjaminhe@nvidia.com

**Keywords:** aneurysm, CFD, blood flow, turbulence model, rheology

## Abstract

An aneurysm is a vascular malformation that can be classified according to its location (cerebral, aortic) or shape (saccular, fusiform, and mycotic). Recently, the study of blood flow interaction with aneurysms has gained attention from physicians and engineers. Shear stresses, oscillatory shear index (OSI), gradient oscillatory number (GON), and residence time have been used as variables to describe the hemodynamics as well as the origin and evolution of aneurysms. However, the causes and hemodynamic conditions that promote their growth are still under debate. The present work presents numerical simulations of three types of aneurysms: two aortic and one cerebral. Simulation results showed that the blood rheology is not relevant for aortic aneurysms. However, for the cerebral aneurysm case, blood rheology could play a relevant role in the hemodynamics. The evaluated turbulence models showed equivalent results in both cases. Lastly, a simulation considering the fluid–structure interaction (FSI) showed that this phenomenon is the dominant factor for aneurysm simulation.

## 1. Introduction

An aneurysm is a vascular malformation that can be classified according to its location (cerebral, aortic) or shape (saccular, fusiform, and mycotic). The study of blood flow interaction with aneurysms has gained attention from physicians and engineers in recent years [1]. Computational fluid dynamics (CFD) has allowed the use of patient-specific aneurysm geometries [2], substituting the numerical simulation and experimental measurements on idealized models [3].

Furthermore, CFD allows the evaluation of hemodynamics parameters, like wall shear stresses (WSS) and oscillatory shear index (OSI), as variables to describe the origin and evolution of aneurysms [4]. In recent years, WSS and OSI, in combination with morphological parameters (size, shape, and location), have been linked with aneurysm rupture risks [5]. Therefore, it is of importance to assure that the numerical results adequately describe the biological part. Properly selecting laminar or turbulent flow and the rheological model of the blood are factors that have a great impact on the numerical solutions.

The Reynolds number of blood flow in intracranial aneurysms is around 400, and the mean value is 600 for abdominal aneurysms [6]. Therefore, according to the classical pipe theory, the flow is laminar. Lee et al. [7] performed numerical simulations considering laminar flow to evaluate the size and shape of the aneurysm. Han et al. [8] also employed laminar flow in patient-specific cerebral aneurysms for rapid evaluation and treatment planning. However, Poelma et al. [9] found that the flow in aneurysms is turbulent due the oscillatory nature of the blood flow. Moradicheghamahi et al. [10] found that turbulent models overestimated the time-averaged wall shear stress in stenosed carotid arteries, estimating less damage in the wall than the real value.

On the other hand, it is well-known that blood is a non-Newtonian fluid, and its rheological behavior depends on the diameter of the vessel, the Fåhraeus–Lindqvist effect [11]. According to the characteristic length of an abdominal aneurysm, the blood can be considered as a Newtonian fluid. For intracranial aneurysms, the mean artery diameter is around 1 [mm], and the rheology is in the limit between Newtonian and non-Newtonian. Abbasian et al. [12] studied the influence of rheological models in atherosclerotic coronary arteries, considering the most common models used in numerical simulations, that is, the Casson, Carreau, and power law. They found that Carreau, modified Casson, and Quemada viscosity blood models were the most accurate. However, the results shown that blood flow can be considered laminar in some sections with high WSS. Abugattas et al. [13] analyzed the influence of the power law, Cross, and Carreau–Yasuda models in the carotid artery flow pattern; their results shown that the power law estimates lower WSS in comparison to the other models.

A third effect relevant in the blood flow simulation in aneurysms is the elastic properties of the wall. Most of the numerical simulations employed rigid wall considerations [14,15], but the cerebral arteries are elastic. Therefore, the fluid–structure interaction (FSI) must be considered [16,17]. According to Moradicheghamahi et al. [10], the elastic walls have a greater effect on the CFD analysis than the rheology or laminar/turbulent flow.

The use of turbulent and rheological models and the rigidness or elasticity of the artery wall to numerically describe the flow in intracranial aneurysms is still under debate. In the present work, a comparison of a turbulent model (k−ϵ model) and laminar model is carried out for three aneurysms, two aortic aneurysms (AAA1 and AAA2) and a cerebral aneurysm (IA). Four rheological models are evaluated: Newtonian, power law, Carreau, and Casson. Finally, the rigid wall solutions are compared with the aneurysms with elastic walls. WSS, time-averaged wall shear stress (TAWSS), OSI, relative residence time (RRT), and gradient oscillatory number (GON) were calculated for each case, according to the definitions given by [18], and the results were compared with the laminar case.

## 2. Materials and Methods

Numerical simulations were performed to assess the influence of blood rheology, turbulence models, and wall elasticity on the fluid dynamics in aortic and intracranial aneurysms. Patient-specific cases were employed to rebuild the geometries.

### 2.1. Governing Equations

A CFD simulation implemented in OpenFOAM [19] was used to solve the mass (Equation (1)) and momentum conservation (Equation (2)) equations under the assumptions that blood is a homogeneous incompressible fluid and that the fluid flow is isothermal. Therefore, the Navier–Stokes equations can be written as

(1) $$\frac{\partial }{\partial t}\left(\rho \right)+\frac{\partial }{\partial x_{i}}\left(\rho u_{i}\right)=0$$

(2) $$\frac{\partial }{\partial t}\rho u_{i}+u_{j}\frac{\partial }{\partial x_{j}}\rho u_{i}=-\frac{\partial p}{\partial x_{i}}+\frac{\partial \tau_{ij}}{\partial x_{j}}$$

where $u_{i}$ is the velocity vector, ρ is density, *t* is time, and *p* is the pressure field. To evaluate the influence of blood rheology, the Newtonian, Carreau, Casson, and power law rheological models were used to describe the behavior of blood viscosity (μ) in two abdominal aortic aneurysms (AAAs) and an intracranial aneurysm (IA).

Newtonian fluid is characterized by a linear relationship between shear stress and shear rate. The relation is $\tau_{ij}=-\mu {\dot{\gamma }}_{ij}$, where $\tau_{ij}$ is the stress tensor, μ is the fluid viscosity, and ${\dot{\gamma }}_{ij}=▿u+\left({▿u}^{T}\right)$ is the rate-of-strain tensor. On the other hand, the simplest non-Newtonian model is the power law, where the relation between the shear stress and shear rate is non-linear, defined as

(3) $$\mu_{eff}=K{\dot{\gamma }}_{ij}^{\left(n-1\right)}$$

where *n* is the power law index, and *K* is the consistency index. For n>1, Equation (3) describes a dilatant fluid; meanwhile, for n<1, a pseudoplastic or shear-thinning fluid is obtained. In shear-thinning fluids, like blood, the shear rate increases while the apparent viscosity decreases. The drawback of this model is that it can overestimate or underestimate the viscosity at very high or very low values of strain rates, respectively, which may not have a physical sense.

In order to describe properly the shear-thinning blood behavior, two models had been defined. The first one is the Cassson model, given by

(4) $$\mu_{eff}={\left(\sqrt{m}+\sqrt{\frac{\tau_{c}}{{\dot{\gamma }}_{ij}}}\right)}^{2}$$

where $\tau_{c}$ is interpreted as the yield stress and *m* is the viscosity consistency. The second one is the Carreau model, which provides a smooth transition between the viscosity limit values, given by the following equation:

(5) $$\mu_{eff}=\mu_{\infty }+\left(\mu_{0}-\mu_{\infty }\right){\left[1+{\left(\lambda {\dot{\gamma }}_{ij}\right)}^{2}\right]}^{\frac{n-1}{2}}$$

$\mu_{\infty }$ and $\mu_{0}$ are the minimum and maximum viscosity limit values obtained experimentally. $\mu_{0}$ is the viscosity value when the blood is not subjected to shear stress, and $\mu_{\infty }$ is the minimum viscosity value reached after a large strain rate (>100) is applied. Commonly, the $\mu_{\infty }$ value is used as the Newtonian constant value. The non-Newtonian behavior is described by the time λ and the exponent *n*, where n<1. The parameters used in the rheological models are shown in the Table 1.

### 2.2. Transport Equations for the Standard k−ϵ Model

The k−ϵ model was used to study the influence of turbulence on the hemodynamics in aneurysms. The model solves the equations of kinetic energy (6) and the dissipation of kinetic energy (7) to determine the turbulent viscosity (8).

(6) $$\frac{\partial }{\partial t}\left(\rho k\right)+\frac{\partial }{\partial x_{i}}\left(\rho ku_{i}\right)=\frac{\partial }{\partial x_{j}}\left[\left(\mu +\frac{\mu_{t}}{\sigma_{k}}\right)\frac{\partial k}{\partial x_{j}}\right]+P_{k}-\rho \epsilon$$

(7) $$\frac{\partial }{\partial t}\left(\rho \epsilon \right)+\frac{\partial }{\partial x_{i}}\left(\rho \epsilon u_{i}\right)=\frac{\partial }{\partial x_{j}}\left[\left(\mu +\frac{\mu_{t}}{\sigma_{\epsilon }}\right)\frac{\partial \epsilon }{\partial x_{j}}\right]+C_{1\epsilon }\frac{\epsilon }{k}P_{k}-C_{2\epsilon }\rho \frac{\epsilon^{2}}{k}$$

(8) $$\mu_{t}=\rho C_{\mu }\frac{k^{2}}{\epsilon }$$

where *k* is the kinetic energy, ϵ is the dissipation of *k*, $P_{k}$ is the generation of kinetic turbulence by the mean velocity gradients, $\sigma_{k}$, $\sigma_{\epsilon }$, $C_{1\epsilon }$ and $C_{2\epsilon }$ are constants. The constant values have the following default values: $\sigma_{k}=1.0$, $\sigma_{\epsilon }=1.3$, $C_{1\epsilon }=1.44$, $C_{2\epsilon }=1.92$, $C_{\mu }=0.09$, according to [10].

### 2.3. Fluid–Solid Interaction (FSI)

In order to study the effect of the rigid artery wall simplification on the results, an FSI simulation was developed in aortic aneurysm AAA2 where the linear elastic model was chosen to model the elastic behavior of the artery wall. Bazilevs et al. [24] and Torii et al. [25] concluded that the linear elastic model is a good approximation for modeling the wall elasticity, after comparing different wall models.

The mechanical properties of the wall tissue are considered uniform, and were based on Isaksen et al. [26]: $\rho^{s}=1000$ kg/m^3^, E=1 MPa, $\nu^{s}=0.45$, and wall thickness of 2 mm.

The solid momentum equation with the linear elastic consideration is written as follows:

(9) $$\rho^{s}\frac{\partial^{2}\delta }{\partial t^{2}}-\nabla \cdot \left[\left(2\mu^{s}+\lambda^{s}\right)\nabla \delta \right]=\nabla \cdot \left[-\left(\mu^{s}+\lambda^{s}\right)\nabla \delta +\mu^{s}\nabla^{T}\delta +\lambda^{s}tr\left(\nabla \delta \right)I\right]$$

where $\rho^{s}$ is density, δ is the displacement tensor, $\mu^{s}$ and $\lambda^{s}$ are the Lame constants, and *I* is the second-order tensor.

### 2.4. Boundary Conditions

For the reological and turbulence analysis, the artery wall was assumed to be rigid, and the non-slip boundary condition was applied. A pulsatile flow was defined at the inlet, using the waveform from Banerje et al. [27], with a mean Reynolds value of 600 in AAA, and 400 in IA. A pressure value of 0 Pa was used in the outlet branches. Density was considered constant with a 1056 kg/m^3^ value. Viscosity (μ) depends on the rheological model. Six cardiac cycles were used to perform the transient simulations, as the condition for the existence of the periodic solution, and the results of the last cycle were analyzed.

For the FSI case, the same inlet and outlet boundary conditions of the rigid wall case were used. The displacement of the fluid and solid interfaces must be the same (Equation (10)), and the traction in both domains must be in equilibrium (Equation (11)). Furthermore, the fluid obeys the non-slip condition on the solid wall. The solid sides are fixed, with zero displacement, and a pressure of 0 Pa was imposed on the external wall.

(10) $$\delta_{s}=\delta_{f}$$

(11) $$\sigma_{s}\cdot {\hat{n}}_{s}=\sigma_{f}\cdot {\hat{n}}_{f}$$

The time step was variable to maintain a Courant number less than 0.1, for numerical stability. A second-order scheme was used to discretize the governing equations.

### 2.5. 3D Model

The patient-specific aneurysm geometries were extracted from digital subtraction angiography (DSA) images, using the open-source software 3D Slicer. A consent form and an explanation of the research performed on these data were provided to the participants. All participants provided their informed written consent. In addition, the institutional review board of the University of Colima approved this study.

**Figure 1:**
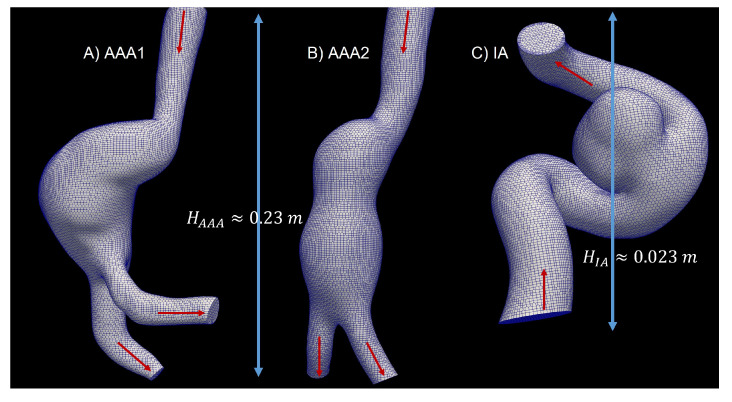
Aneurysm geometries, mean size, flow direction (red arrows) and meshes under study.

To isolate the area of the aneurysm and the parent vessel, the threshold tool available in 3D Slicer was used to distinguish blood from the rest of the tissues. The artery and the tissue around the artery were removed, and the 3D model was exported to STL format. This result was used to mesh the analyzed geometry in the simulation. The aortic aneurysms (AAA1 and AAA2) and cerebral aneurysm (IA) are shown in Figure 1.

### 2.6. Grid Generation

Mesh generation of the computational models was performed using the OpenFOAM’s tool snappyHexMesh. The mesh was hexahedral dominant, and a prismatic boundary layer was implemented to capture accurately the influence of the wall and the WSS.

To define the mesh size, the criterion of having at least 25 even cells that covered the diameter of the artery was used. The cell size for intracranial aneurysms was sought to be $2\times 10^{-4}$ m, with three boundary layers, where the first cell had a size of $1.4\times 10^{-4}$ m. For aortic aneurysms, the cell size was $1\times 10^{-3}$ m, with three boundary layers, where the first element was $5\times 10^{-4}$ m. $Y^{+}$ values were reviewed for both types of aneurysms, and the average peak systole for the intracranial aneurysm was 0.9, while for the abdominal aorta, it was 0.7, achieving lower values during the rest of the pulsating signal.

**Figure 2:**
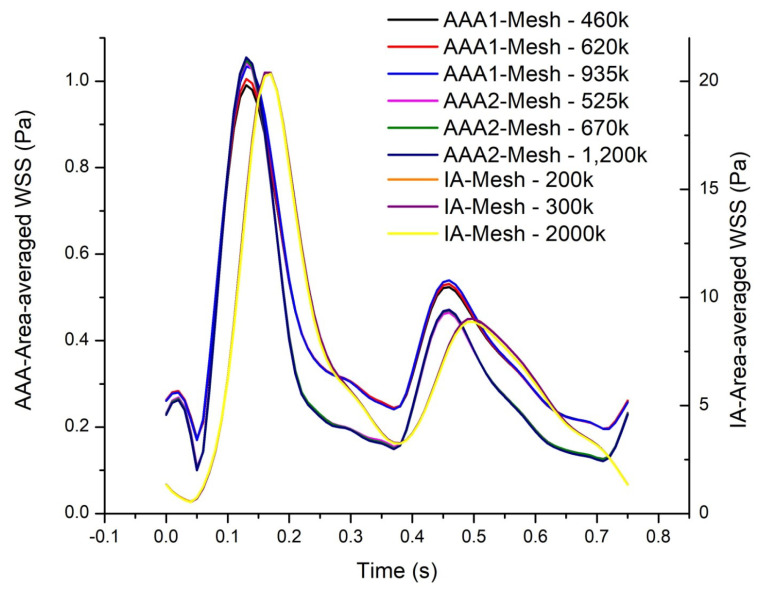
Mesh independence test of the area-averaged WSS as a function of time for the whole surface for all cases.

Three cases were studied: two abdominal aortic aneurysms (AAA1 and AAA2) and an intracranial aneurysm (IA) (Figure 1). A mesh independence test was developed in each model. Three different meshes were created for the abdominal aortic aneurysm (AAA1), with 460k (coarse), 620k (medium), and 935k (fine) elements. In order to compare the results of these three meshes, the TAWSS of the last cardiac cycle was analyzed and plotted (Figure 2). The maximum difference of the fine and coarse mesh was 5 %, and with respect to the medium mesh, the maximum difference was 3 %. In the second abdominal aortic aneurysm (AAA2), the meshes studied comprised 525k (coarse), 670k (medium), and 1200k (fine) elements. The maximum difference of area-averaged WSS between the fine and the coarse meshes was 8 %, and with respect to the medium mesh, the maximum difference was 3%. For the intracranial aneurysm (IA), the meshes studied comprised 200k (coarse), 300k (medium), and 2000k (fine) elements. The maximum differences comparing the fine mesh with the coarse and medium mesh were 7% and 4%, respectively. The meshes selected for the rheological and turbulence analysis were the medium-resolution meshes for all models, which had a percentage error less than 5%.

A wall turbulence model was employed. For the three cases, $Y^{+}=2$ was the maximum value obtained, while the maximum average value was $Y^{+}=0.25$. Therefore, the turbulent boundary layer can be resolved correctly. On the other hand, the mesh for the solid artery wall in the FSI study was achieved by extruding the fluid wall, with a constant thickness of 2 mm, built by 5 layers with an expansion ratio of 1.2. This method allows the match between the fluid and solid meshes.

The rheology and turbulence studies were carried out using the PisoFOAM solver from OpenFOAM v8.0, while the FSI analysis was performed with Foam-Extend v4.0 using the Solids4FOAM and Swak4FOAM tools. Given that the optimization of the time step depends on the pulsating signal, an adjustable time step was used to keep a Courant number less than 0.2 to guarantee convergence. To test the numerical solution, a comparison with the results obtained by Epshtein and Korin [3] with maximum error of 5% validated the numerical procedure.

## 3. Results

Velocity, WSS, TAWSS, OSI, RRT, and GON were calculated according to the definitions given by [18]. The WSS was determined for systole and diastole; meanwhile, the other parameters were calculated for the last cardiac cycle. The most relevant figures are shown in this section; however, for the interested reader, the repository available in [28] provides the results for all cases.

**Figure 3:**
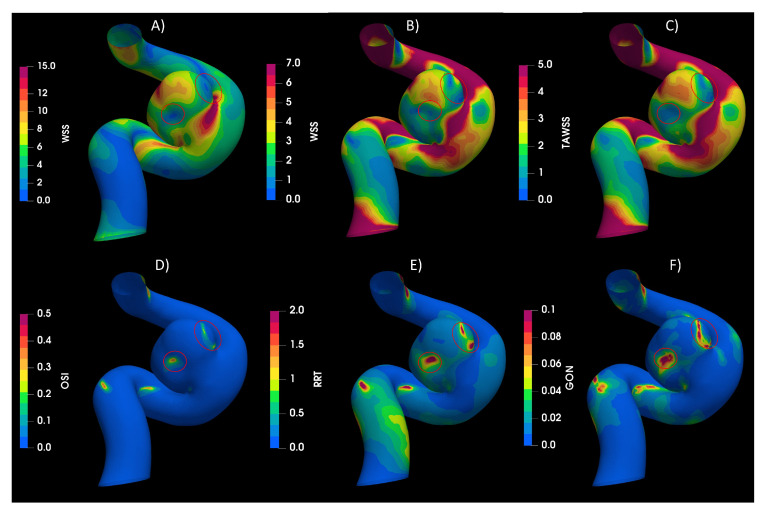
Hemodynamic indices studied in IA. (**A**) WSS in peak diastole, (**B**) WSS in peak systole, (**C**) TAWSS, (**D**) OSI, (**E**) RRT, and (**F**) GON. The circled areas shown the zone with the maximum value for each variable.

The IA’s indices showed high values of WSS for systole, being the dominant effect on the TAWSS. The circled regions in Figure 3 indicate the zones of low WSS and high values of OSI, RRT, and GON. The IA results, which have an aspect ratio (AR) of 1.13, show that the blood flow goes upstream through the parent artery (ICA), impacting the distal side of the neck. Then the flow goes towards the aneurysm dome and finally returns to the parent vessel by the proximal side of the neck, completing one vortex, as described by Munarriz et al. [29]. The highest value of WSS happened in the distal neck because of the impinging flow, while the values of WSS in the dome depend on the velocity distribution in the sac. Meng et al. [30] proposed that the growth and rupture of an aneurysm is associated with high or low values of WSS, depending on the aneurysm shape and flow behavior. The numerical results shows that the IA belongs to the slow recirculation class, according to [30]. High values of OSI and low values of WSS can trigger inflammatory-cell-mediated destructive remodeling that leads to a large, thick wall aneurysm phenotype.

**Figure 4:**
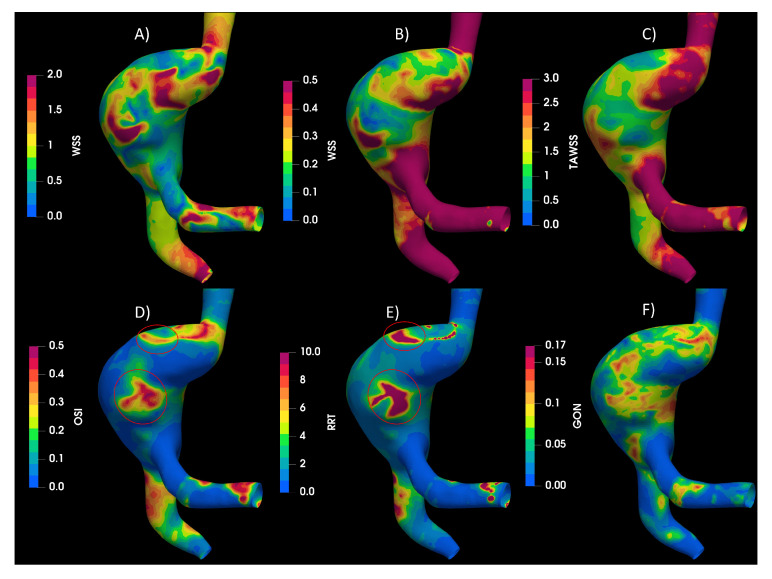
Hemodynamic indices studied in AAA1. (**A**) WSS in peak Diastole, (**B**) WSS in peak systole (**C**) TAWSS, (**D**) OSI, (**E**) RRT and (**F**) GON.

In the aortic aneurysms AAA1 and AAA2 (Figure 4) the flow descends through the aorta to the aneurysm zone, where multiple vortices form; furthermore, the results exhibit some areas with recirculation and stagnation flow. The circled regions meet the condition of low WSS and high OSI and RRT. Almost all the proposed indices points to regions with high probability of destructive wall remodeling according to [30], except GON, that presented zones of high values in different parts of the aneurysm and its influence was not clear.

### 3.1. Rheology

The strain rate results in Table 2 were calculated at the artery wall, at the aneurysm wall, at the blood volume in the artery (artery body), and at the blood volume in the aneurysm (aneurysm body). The strain rate values at the wall for IA were significantly higher than the AAA cases. As was expected, the strain rates in the fluid were lower than the values at the wall; however, IA cases presented values an order of magnitude larger than the AAA. The values are relevant in order to determine the rheology influence on the flow behaviour.

The IA strain rate values were higher than the AAAs’ strain rate values. The IA strain rate values in the artery and aneurysm wall were in the order of $10^{3}$, whereas the strain rate values in the total artery body and only the aneurysm body were higher than $10^{2}$. The AAAs’ values in the wall were near 100, which is the critical strain rate value where the viscosity starts to behave like a constant Newtonian value in the non-Newtonian models. Values in the body far away from the wall are low, and differences between Newtonian and non-Newtonian models were expected.

The results of the rheological analysis in intracranial aneurysms show minimal differences between the models when considering the different indices under study, with similar qualitative results between the Newtonian and non-Newtonian models. This can be explained because the strain rate is larger than 100 in the body and the aneurysm wall, as shown in Table 2. The AAAs’ results show larger differences than IA in the studied indices. AAA2 was the most critical case, showing larger differences that could be attributed to low values of strain rate, even in the wall.

**Figure 5:**
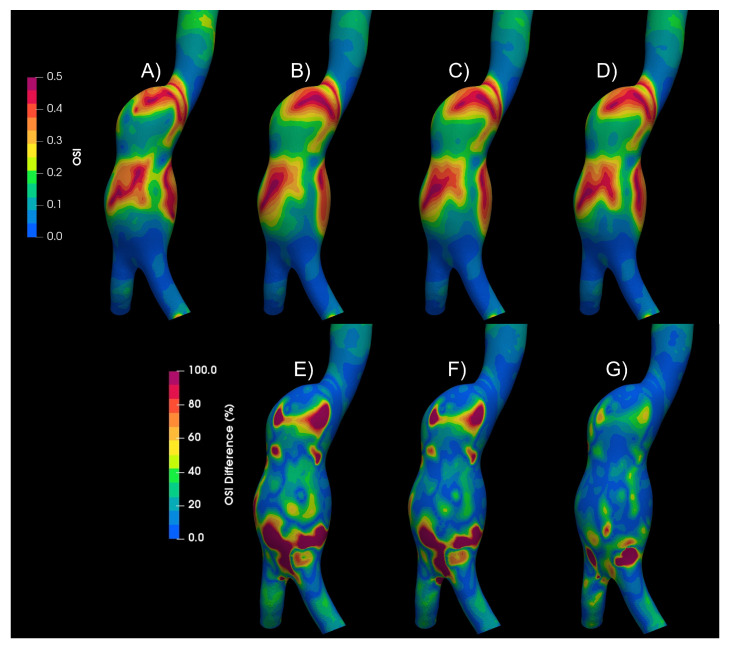
OSI results of Newtonian and non-Newtonian models. (**A**) Newtonian, (**B**) Crreau, (**C**) Casson, (**D**) power law, (**E**) Newtonian–Carreau differences, (**F**) Newtonian–Casson differences, (**G**) Newtonian–power law differences.

Figure 5 shows the OSI contours for the Newtonian and the non-Newtonian models. Qualitative differences in the patterns can be observed. Below the OSI contours (A to D), the differences of each of the non-Newtonian vs. Newtonian models are shown (E to G), and some areas with differences of up to 100% can be observed. Similar differences were found in WSS, TAWSS, RRT, and GON repository.

**Figure 6:**
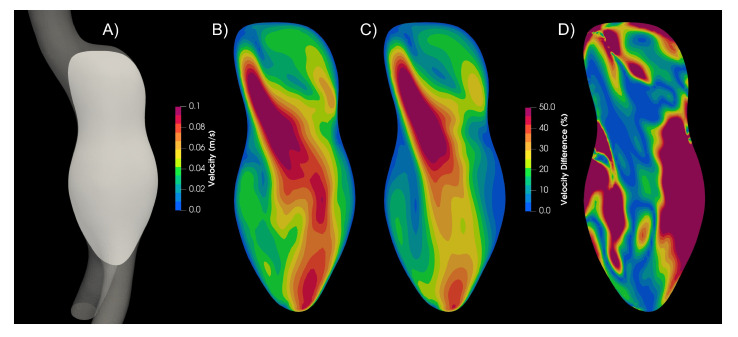
Velocity plane contour. (**A**) Plane, (**B**) Newtonian model, (**C**) Carreau model, (**D**) Newtonian–Carreau differences.

In aortic aneurysms, far from the wall, the strain rate values are low, and the viscosity values differ from the Newtonian case. Therefore, the velocity profiles differ between Newtonian and non-Newtonian models. In Figure 6, the magnitude of velocity of the Newtonian and Carreau models are compared. The Newtonian model presents higher velocity values, and 25% of the plane area had a 50% difference or higher.

### 3.2. Turbulence

Turbulence is a complex phenomenon characterized by chaotic and unstable flows. It is known that, for flow in pipes, the critical Reynolds number (Re) is ≈2300. For values below Re, laminar flow can be considered, and for values above Re, turbulent flow starts to form. In the present study, a pulsatile flow was taken into account [27], and according to the physiological conditions, a mean Reynolds number ${Re}_{mean}\approx 600$ was used for AAA, with variations of ${Re}_{min}\approx 35$ to ${Re}_{max}\approx 1367$, with a Womersley number Wo≈20. For IA, the following data were taken into account: ${Re}_{mean}\approx 400$, ${Re}_{min}\approx 23$, ${Re}_{max}\approx 911$, and Wo≈4.

According to the classical theory in pipes, the blood flow in the aneurysms is laminar. However, Poelma et al. found differences in the velocity fields between the cycles under analysis Poelma et al. [9]. These changes are a characteristic of turbulent flows. The geometry of the aneurysms and the pulsatile nature of the flow are perturbations of the laminar flow and could modify the critical Reynolds number. Furthermore, the deceleration in the cardiac cycle is a destabilizing factor of the laminar flow; therefore, Hershey and Im [31] and NEREM and SEED [32] concluded that the critical Reynolds number is inversely proportional to the Womersley number.

**Figure 7:**
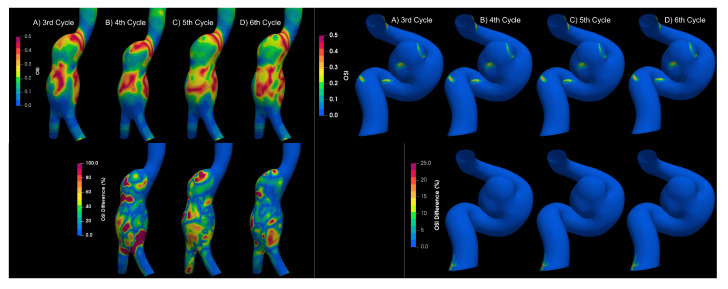
OSI results for different cycles for aortic and intracranial aneurysms.

To determine if the flow is laminar or turbulent, the results of four cardiac cycles were compared for both types of aneurysms. Differences between each cardiac cycle were found for aortic aneurysms, as shown in Figure 7, which is a characteristic of turbulent flows Poelma et al. [9]. On the other hand, for intracanial aneurysms, no significant changes were observed between cardiac cycles; therefore, the flow in IA can be considered as laminar.

**Figure 8:**
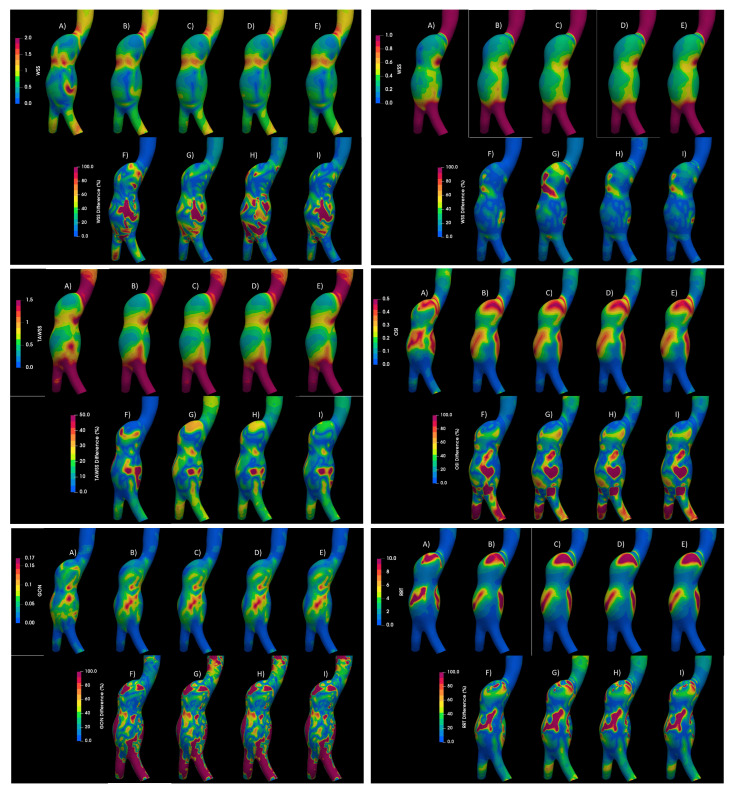
Hemodynamic parameters under study for (**A**) Laminar—Newtonian, (**B**) k−ϵ—Newtonian, (**C**) k−ϵ—Carreau, (**D**) k−ϵ—Casson, (**E**) k−ϵ—power law. (**F**) Differences between Laminar—Newtonian and k−ϵ—Newtonian, (**G**) differences between Laminar—Newtonian and k−ϵ—Carreau, (**H**) differences between Laminar—Newtonian and k−ϵ—Casson, (**I**) differences between Laminar—Newtonian and k−ϵ—power law.

**Figure 9:**
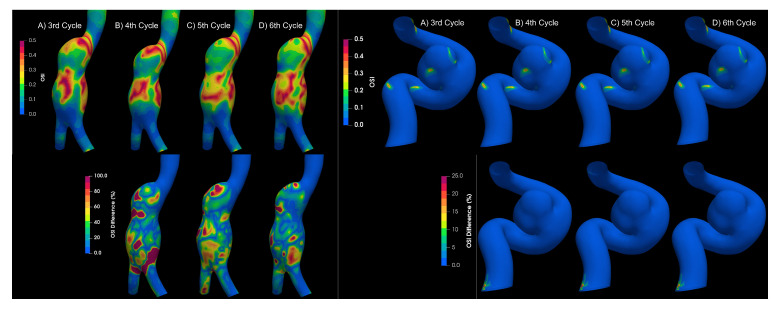
Newtonian value of OSI at different cycles.

**Figure 10:**
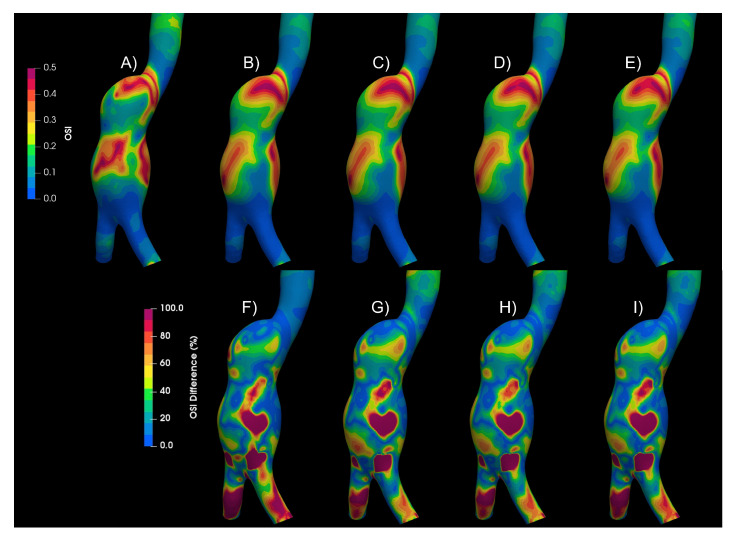
OSI results of Newtonian and non-Newtonian models, comparing turbulence models. (**A**) Newtonian laminar, (**B**) Newtonian KE, (**C**) Carreau KE, (**D**) Casson KE, (**E**) power law—KE, (**F**) Newtonian laminar—Newtonian KE differences, (**G**) Newtonian laminar—Carreau KE differences, (**H**) Newtonian laminar—Casson KE differences, (**I**) Newtonian laminar—power law KE differences.

Numerical simulations for both types of aneurysms were performed considering laminar flow and Newtonian model, turbulent flow (using the K-epsilon model), and four rheological fluids: Newtonian, Carreau, Casson, and power law. Figure 8 shows the WSS, TAWSS, OSI, GON, and RRT for the aortic aneurysm (AAA2) and the differences between them. The areas where the largest differences occur between a laminar and a turbulent flow are circled in the figure. Differences exceeding 100% of the laminar value were found in large areas of the aneurysm, Figure 9 and Figure 10, which overestimate the WSS, and consequently the TAWSS (lower part of the aneurysm), promoting changes in the patterns of OSI, GON, and RRT in the central part of the aneurysm. Small differences (less than 5%) were obtained between the rheological models using the K-epsilon model, suggesting that the turbulent models are more relevant than the rheological characterization of the flow. This same behavior was obtained for the second aortic aneurysm (AAA1). Meanwhile, for the intracranial aneurysm (IA), no differences between the rheological models nor laminar or turbulent flow were found.

### 3.3. Fluid–Solid Interaction (FSI)

**Figure 11:**
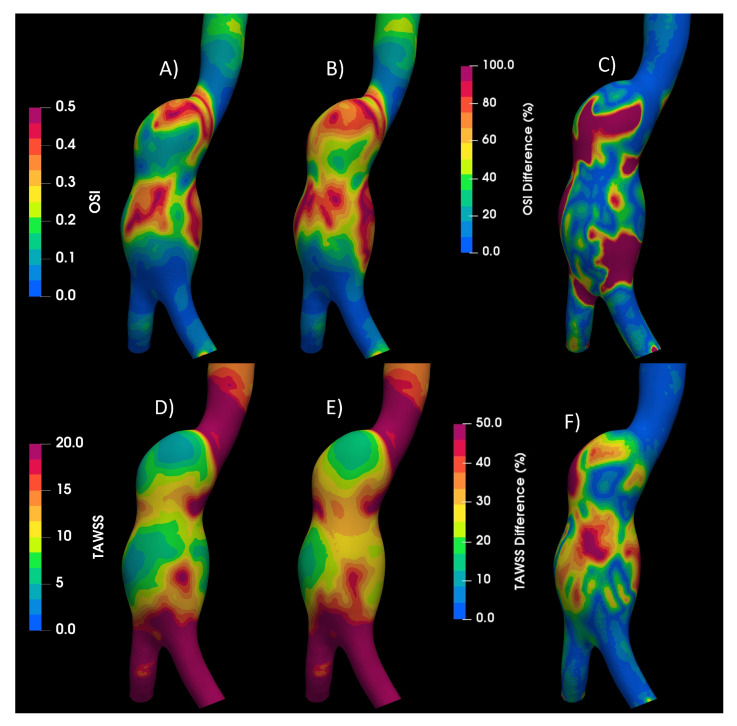
Camparison of rigid and elastic wall. (**A**) OSI values of rigid, laminar, and Newtonian case. (**B**) OSI values of elastic, laminar, and Newtonian case. (**C**) OSI differences between rigid and elastic wall. (**D**) TAWSS values of rigid, laminar, and Newtonian case. (**E**) TAWSS values of elastic, laminar, and Newtonian case. (**F**) TAWSS differences between rigid and elastic wall.

The motion of the elastic arterial wall under the effects of a pulsatile signal presents a different fluid dynamics behavior with respect to the rigid arterial wall. All the indices analyzed present different patterns; the one that presented the greatest difference with respect to these two wall considerations was the GON, where the patterns are completely different, and the average of the differences in the entire area of the aneurysm is 43%, while the next index that had the greatest difference was the WSS during diastole, with 25%. The third place is occupied by the index most related to intracranial aneurysms, the OSI, with a 20% difference, as shown in Figure 11. For the interested reader, the results of all cases are available in [28].

## 4. Discussion

**Figure 12:**
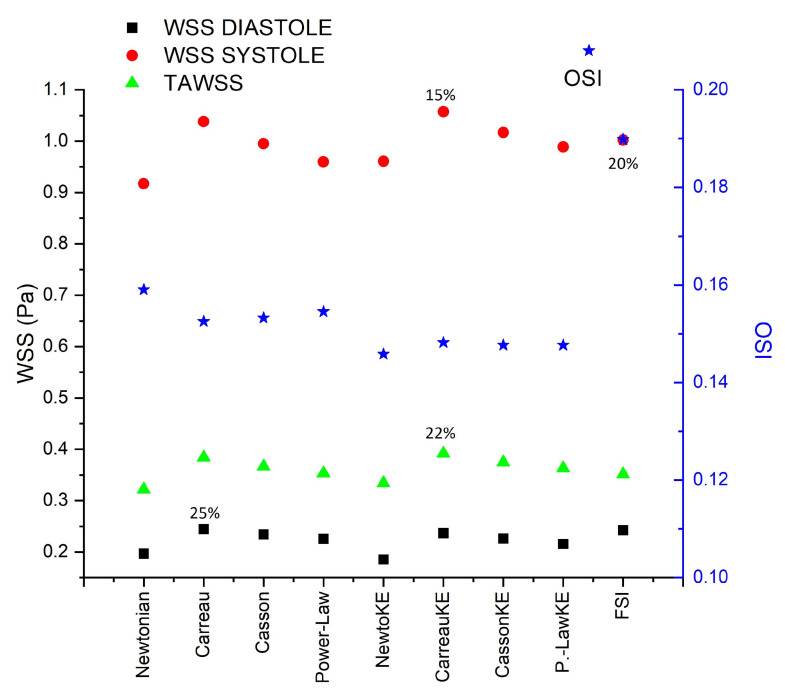
TAWSS in peak diastole, peak systole, TAWSS, and OSI area-averaged (blue star) results in AAA2 under rheological, turbulence, and elastic models studied.

**Figure 13:**
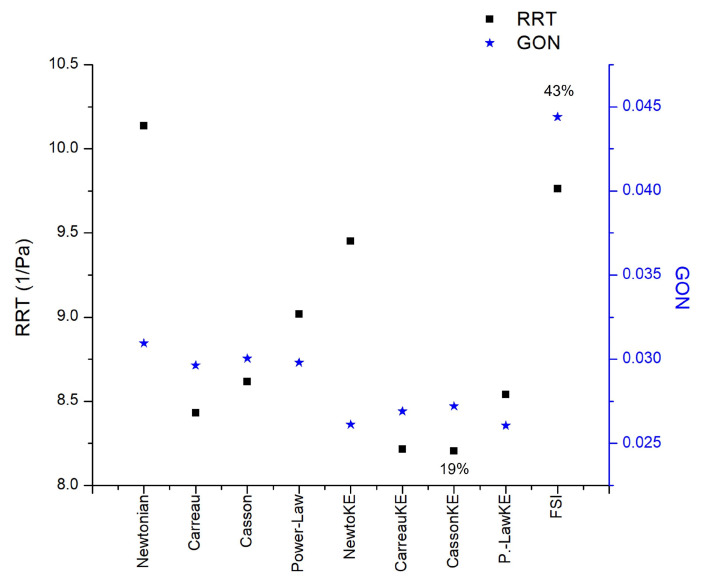
RRT and GON area-averaged results in AAA2 under rheological, turbulence, and elastic models studied.

Laminar and turbulent flow models were compared using four rheological models and rigid or elastic walls to simulate the flow in two aortic aneurysms (AAA) and an intracranial aneurysm (IA). According to Pratumwal et al. [33], the Carreau model is the best to describe the blood rheology; however, for IA the rheology has little influence on the flow behaviour. Meanwhile, for AAA the biggest differences are presented by the Carreau model with respect to the Newtonian model, for WSS (25%) and TAWSS (22%), Figure 12. For RRT the effect of the rheological model is greater than the laminar-turbulent flow, Figure 13.

No differences were found between laminar and turbulent flows for WSS and TAWSS. The turbulent model underestimates the OSI, RRT, and GON in comparison with the laminar flow. Therefore, the flow model is of great relevance for simulating the blood flow in aortic aneurysms. Finally, the most relevant effect to take into consideration for numerical simulations is the rigid–elastic wall assumption.

## Figures and Tables

**Table 1:** Effective viscosity equations of various models with their parameters.

MODEL	PARAMETERS	
Newtonian	μ=0.00345 Pa.s	[20]
Power law	K=0.009267, n=0.828	[21]
Casson	m=0.0031 Pa.s, $\tau_{c}=0.0090$ Pa	[22]
Carreau	$\mu_{0}=0.056$ Pa.s, λ=3.313 s $\mu_{\infty }=0.00345$ Pa.s, n=0.3568	[23]

**Table 2:** Strain rate values for all models.

Strain Rate	AAA1	AAA2	IA
Artery Wall	125	97	1888
Aneurysm Wall	89	73	1480
Artery Body	19	22	502
Aneurysm Body	15	20	388

## Data Availability

The data presented in this study are openly available in Mendeley Data at [https://doi.org/10.17632/rhvvbxwwh4.2] 1 October 2021.

## Abbreviations

The following abbreviations are used in this manuscript:

FSI	Fluid–structure interaction
OSI	Oscillatory shear index
GON	Gradient oscillatory number
CFD	Computational fluid dynamic
AAA	Aortic Aneurysms
IA	Intracranial Aneurysm
WSS	Wall shear stresss
TAWSS	Time-Averaged Wall Shear Stress
RRT	Relative Residence Time
DSA	Digital Substraction Angiography
